# Editorial: Multi-Layered Genome-Wide Association/Prediction in Animals

**DOI:** 10.3389/fgene.2022.877748

**Published:** 2022-04-08

**Authors:** Ruidong Xiang, Lingzhao Fang, Marie-Pierre Sanchez, Hao Cheng, Zhe Zhang

**Affiliations:** ^1^ Faculty of Veterinary and Agricultural Science, The University of Melbourne, Parkville, VIC, Australia; ^2^ Agriculture Victoria, AgriBio, Centre for AgriBiosciences, Bundoora, VIC, Australia; ^3^ MRC Human Genetics Unit at the Institute of Genetics and Cancer, The University of Edinburgh, Edinburgh, United Kingdom; ^4^ INRAE, AgroParisTech, GABI, Université Paris-Saclay, Jouy-en-Josas, France; ^5^ Department of Animal Science, University of California, Davis, Davis, CA, United States; ^6^ College of Animal Science, South China Agricultural University, Guangzhou, China

**Keywords:** multi-omics, biological priors, genome-wide association studies, genomic prediction, genomic selection

DNA mutations are the fundamental source of genomic variations that lead to phenotypic differences between individuals. Genomic variations in a population are usually assayed by single nucleotide polymorphism (SNP) arrays or whole-genome sequencing (WGS) to obtain genotype counts. If phenotypic measurements are also available on genotyped individuals in this population, genotype counts can be statistically linked to phenotypic measurements, i.e., genome-wide association studies (GWAS). Decades of GWAS in humans (Visscher et al., 2017) and animals (Hayes and Daetwyler, 2019) have shown that causal variants for complex traits are largely located at non-coding regions of the genome. This has been further supported by recent human studies of genetic variations with roles in gene regulation, e.g., those that are gene expression quantitative trait loci (eQTL) (Consortium, 2020) are enriched in causal variants of complex traits. Due to the vast availability of data in humans, such as proteomics and metabolomics, great efforts have been invested in the integration of multi-omics information and GWAS results (Hasin et al., 2017). The effort of functional annotation of animal genomes only started recently (Clark et al., 2020), although the size of multi-omics data has been increasing (Liu et al., 2021).

Unlike genomics research in humans, GWAS in animals is usually carried out amongst related individuals with small effective population sizes. This results in many SNPs in high linkage disequilibrium (LD) from a locus being associated with a trait, and it is difficult to distinguish which ones are causal. This is particularly difficult when the GWAS used imputed sequence variants (Hayes and Daetwyler, 2019) where a large number of variants are in very strong LD. Therefore, external information, such as multi-omics datasets independent of GWAS, is needed to pinpoint causal signals. Apart from the use of multi-omics data, multi-trait meta-analyses of GWAS (Xiang et al., 2020; Xiang et al., 2021) and large-scale GWAS of intermediate traits like milk composition (Sanchez et al., 2021) also improve the detection of causal variants. In addition, multi-breed meta-analyses can help to pinpoint causal mutations as LD is conserved over shorter distances across breeds (van den Berg et al., 2020).

The genomic information of domestic animals is used to improve animal breeding. In particular, genomic selection or genomic prediction (GP) (Meuwissen et al., 2001) using genome-wide marker information has greatly benefited animal breeding. GP was primarily designed to use all available markers to estimate genomic breeding values (gEBVs) reflecting the genetic merit of animals. However, the accuracy of GP, approximated as the correlation between gEBVs and phenotype in the validation population is far from being perfect. There are many ways to improve the accuracy of GP and emerging evidence shows that the use of functional information can enhance GP (MacLeod et al., 2016; Xiang et al., 2019; Teng et al., 2020). With the growing size of functional genomics data, it is anticipated that functional genomics priors will be routinely integrated into GP to improve its accuracy. This will then need the development of suitable methodologies that effectively fuse multi-omics data together with the genotype-phenotype association analysis in the GP model (Cheng et al., 2021).

The ‘Multi-layered Genome-wide Association and Prediction in Animals’ research topic intends to collect high-quality articles on the emerging area of integrating multi-omics datasets into GWAS and GP. In its conclusion, 9 articles from 58 authors have been collected, ranging from data generation, integrative analysis of multi-omics with GWAS and GP, and new method development across multiple domestic species.

Developing new methods for the integrative analysis of multi-omics and GWAS/GP is one of the key research areas in genetics. Due to flexibility in incorporating priors in the model, several new Bayesian methods have been proposed, including BayesHP and BayesHE (Shi et al.) that incorporate “global-local” shrinkage priors, and multi-class Bayesian Alphabet methods (Wang et al.) that incorporate biological information into multi-trait Bayesian analysis. The application of these methods into simulated and real data supports that incorporating biological priors into GP training improves its accuracy.

GWAS or GP using WGS is another emerging area. Due to high costs of in-depth WGS, there is a new shift toward using low-pass WGS which provides cost-effective options for GWAS or GP to use millions of sequence variants. By analyzing simulated data, Deng et al. show that imputation using low-pass WGS is more accurate than using SNP arrays. This was also found by Zhao et al. where real low-pass WGS from donkeys were generated, analyzed, and applied to GP.

GWAS in animals has been largely used to dissect causative loci associated with complex traits. Jiang et al. present such an effort in detecting loci associated with body size in Hu sheep. Also, Yang et al. identified loci associated with meat production in chicken. Apart from the standard linear mixed model, GWAS can also be carried out using single-step Bayesian regression, and Naserkheil et al. present such an effort in identifying loci associated with meat production traits of beef cattle.

In fact, loci prioritized by GWAS may be used as biological priors to enhance GP. However, Gebreyesus et al. found that adding GWAS-prioritized variants had no improvement in GP for survival traits of dairy cattle which have very low heritability estimates. This emphasizes that more studies are needed in this area. Other lowly heritable but important traits in cattle included female fertility. Chen et al. found that accounting for sire genetic effects improves the genetic evaluation of fertility of Holstein cows.

In conclusion, integrating multi-omics data with GWAS and GP in animals is an important and emerging research area in livestock genomics. We anticipate that the development and application of efficient methods, increased use of WGS, and integration of more types of multi-omics data will be future directions of this area. Understanding how DNA mutations shape complex traits not only furthers our understanding of biology, but also provides practical benefits in animal breeding.
